# Supplementary material for: Crystal and solution structures reveal oligomerization of individual capsid homology domains of Drosophila Arc
Source: PLoS One. 2021 May 14;16(5):e0251459. doi: 10.1371/journal.pone.0251459 (PMC8121366; doi:10.1371/journal.pone.0251459)
Supplement: S1 Table — (PDF) [file pone.0251459.s001.pdf]

S1 Table. The expression constructs used in the current study.

Construct	Uniprot entry	Residues	N-terminal extension
dArc1-NL	Q7K1U0	51-122	SGSG
dArc1-CL	Q7K1U0	123-208	SGSG
dArc2-NL	Q7JV70	38-109	S
dArc2-CL	Q7JV70	110-193	SGSG
hArc-NL	Q7LC44	207-277	GAMG
hArc-CL	Q7LC44	277-370	GAM
dArc1 (NL+CL)	Q7K1U0	51-208	S
dArc2 (NL+CL)	Q7JV70	38-193	-
