# Supplementary material for: Crystal and solution structures reveal oligomerization of individual capsid homology domains of Drosophila Arc
Source: PLoS One. 2021 May 14;16(5):e0251459. doi: 10.1371/journal.pone.0251459 (PMC8121366; doi:10.1371/journal.pone.0251459)
Supplement: S1 Fig — (PDF) [file pone.0251459.s002.pdf]

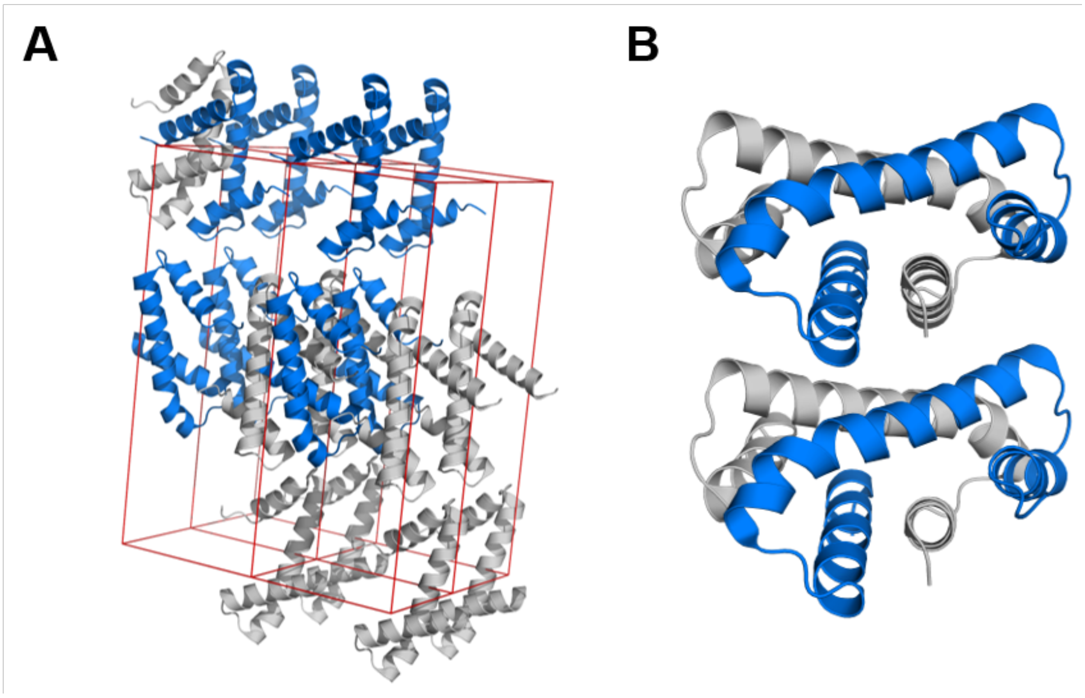


**S1 Fig.** Crystal packing in dArc2-NL. (A) The unit cell. (B) Stacking of dArc2-NL domain-swapped dimers in the lattice.
