# Supplementary figures and images for: Crystal and solution structures reveal oligomerization of individual capsid homology domains of Drosophila Arc
Source: PLoS One. 2021 May 14;16(5):e0251459. doi: 10.1371/journal.pone.0251459 (PMC8121366; doi:10.1371/journal.pone.0251459)

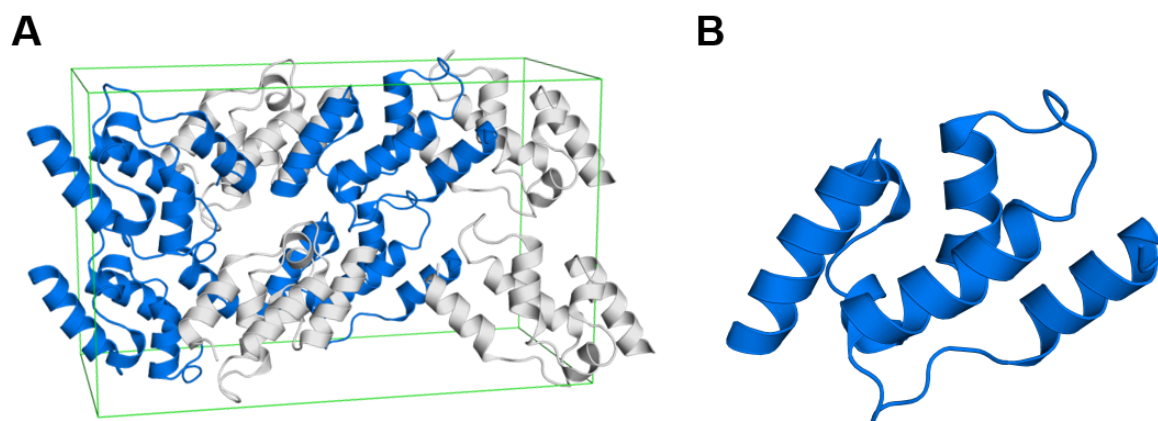

**S2 Fig.** Crystal packing in dArc1-CL. (A) The unit cell. (B) A monomer of dArc1-CL.

Supplement: S2 Fig — (PDF) [file pone.0251459.s003.pdf]
