# Supplementary material for: Crystal and solution structures reveal oligomerization of individual capsid homology domains of Drosophila Arc
Source: PLoS One. 2021 May 14;16(5):e0251459. doi: 10.1371/journal.pone.0251459 (PMC8121366; doi:10.1371/journal.pone.0251459)
Supplement: S3 Fig — (PDF) [file pone.0251459.s004.pdf]

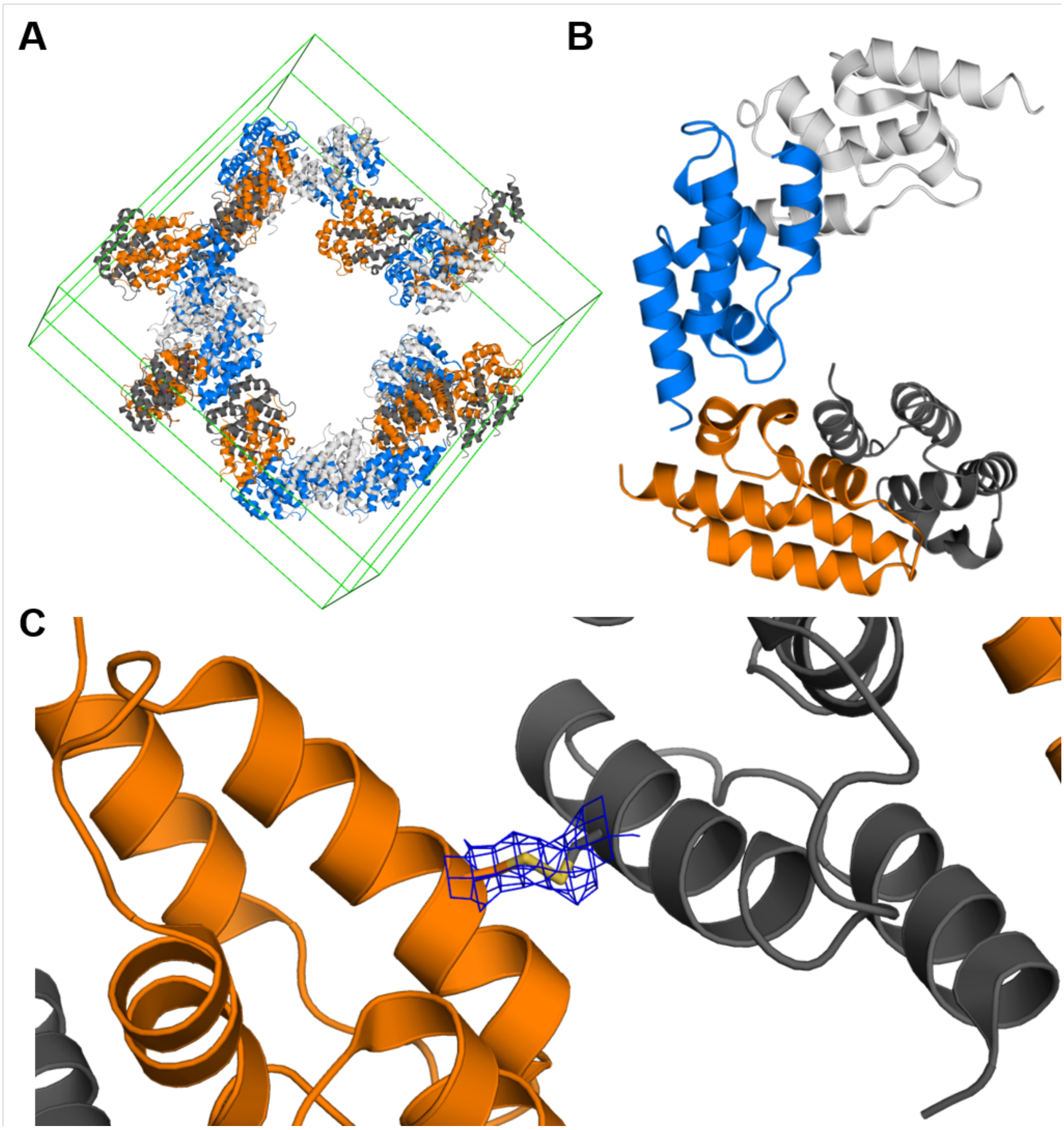

S3 Fig. Crystal packing in dArc2-CL. (A) The unit cell. (B) dArc2-CL is packed as homodimers. (C) In the crystal lattice, dimers are linked to each other *via* a disuplhide bridge.
