# Supplementary material for: Crystal and solution structures reveal oligomerization of individual capsid homology domains of Drosophila Arc
Source: PLoS One. 2021 May 14;16(5):e0251459. doi: 10.1371/journal.pone.0251459 (PMC8121366; doi:10.1371/journal.pone.0251459)
Supplement: S4 Fig — (PDF) [file pone.0251459.s005.pdf]

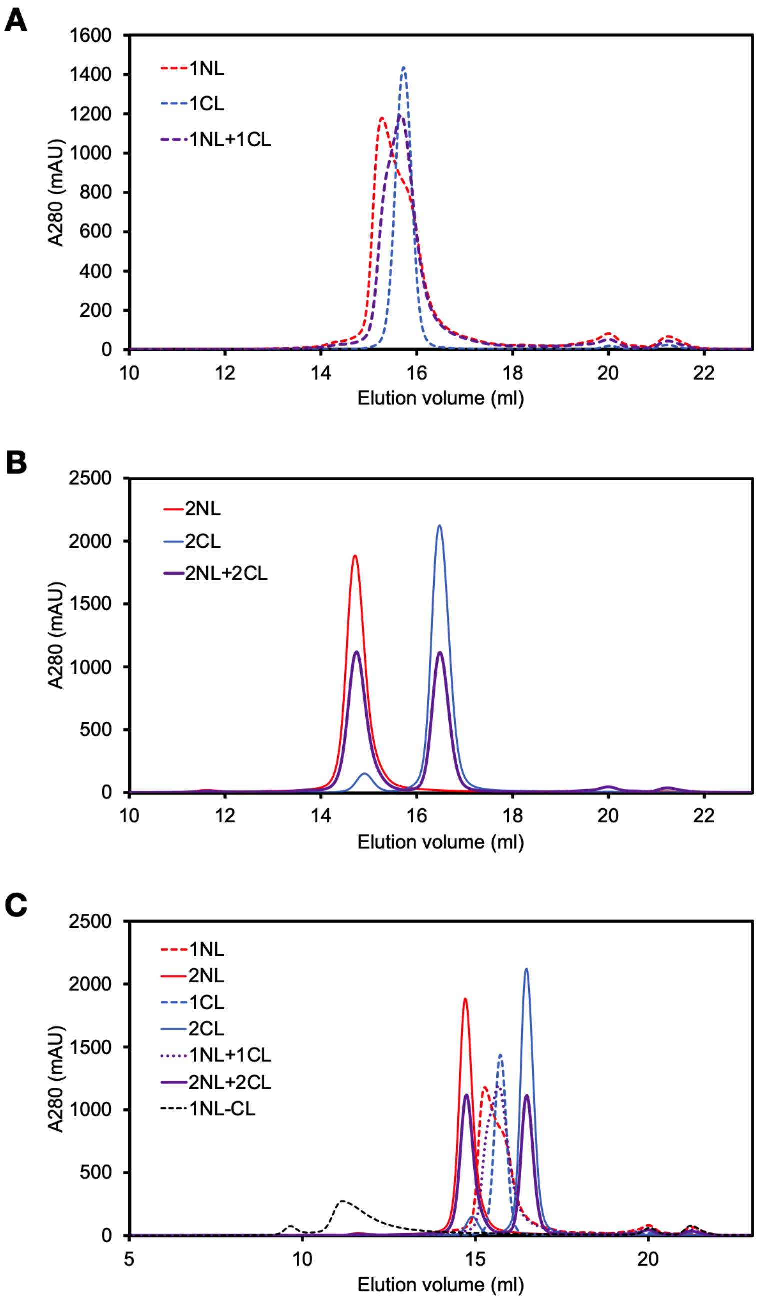

S4 Fig. Mixing of dArc-NL and dArc-CL does not result in larger oligomers. (A) SEC for the dArc1 lobes separately and together. Both dArc1-NL and dArc1-CL are dimeric. (B) SEC for the dArc2 lobes separately and together. dArc2-NL is tetrameric and dArc2-CL dimeric. (C) All plots overlaid, with a SEC run for the dArc1 construct with both lobes added, showing large oligomers.
