# Supplementary material for: Crystal and solution structures reveal oligomerization of individual capsid homology domains of Drosophila Arc
Source: PLoS One. 2021 May 14;16(5):e0251459. doi: 10.1371/journal.pone.0251459 (PMC8121366; doi:10.1371/journal.pone.0251459)
Supplement: S2 File — (PDF) [file pone.0251459.s007.pdf]

Full wwPDB X-ray Structure Validation Report ⓘ

May 22, 2020 – 03:19 pm BST

PDB ID	:	6SID
Title	:	Crystal structure of the C-lobe of drosophila Arc 1 at atomic resolution
Authors	:	Hallin, E.I.; Kursula, P.
Deposited on	:	2019-08-09
Resolution	:	1.05 Å(reported)

This is a Full wwPDB X-ray Structure Validation Report for a publicly released PDB entry.

We welcome your comments at *validation@mail.wwpdb.org*
A user guide is available at
https://www.wwpdb.org/validation/2017/XrayValidationReportHelp
with specific help available everywhere you see the ⓘ symbol.

The following versions of software and data (see references ⓘ) were used in the production of this report:

MolProbity	:	4.02b-467
Xtriage (Phenix)	:	1.13
EDS	:	2.11
Percentile statistics	:	20191225.v01 (using entries in the PDB archive December 25th 2019)
Refmac	:	5.8.0158
CCP4	:	7.0.044 (Gargrove)
Ideal geometry (proteins)	:	Engh & Huber (2001)
Ideal geometry (DNA, RNA)	:	Parkinson et al. (1996)
Validation Pipeline (wwPDB-VP)	:	2.11

1 Overall quality at a glance (i)

The following experimental techniques were used to determine the structure:
X-RAY DIFFRACTION

The reported resolution of this entry is 1.05 Å.

Percentile scores (ranging between 0-100) for global validation metrics of the entry are shown in the following graphic. The table shows the number of entries on which the scores are based.

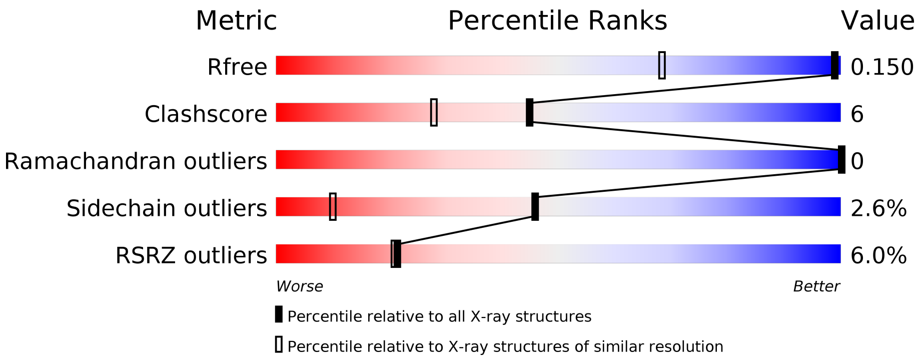

Metric	Whole archive (#Entries)	Similar resolution (#Entries, resolution range(Å))
R_{free}	130704	1202 (1.10-1.02)
Clashscore	141614	1252 (1.10-1.02)
Ramachandran outliers	138981	1204 (1.10-1.02)
Sidechain outliers	138945	1202 (1.10-1.02)
RSRZ outliers	127900	1178 (1.10-1.02)

The table below summarises the geometric issues observed across the polymeric chains and their fit to the electron density. The red, orange, yellow and green segments on the lower bar indicate the fraction of residues that contain outliers for >=3, 2, 1 and 0 types of geometric quality criteria respectively. A grey segment represents the fraction of residues that are not modelled. The numeric value for each fraction is indicated below the corresponding segment, with a dot representing fractions <=5% The upper red bar (where present) indicates the fraction of residues that have poor fit to the electron density. The numeric value is given above the bar.

Mol	Chain	Length	Quality of chain
1	A	90	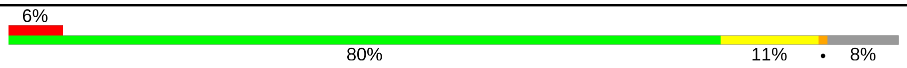

2 Entry composition (i)

There are 2 unique types of molecules in this entry. The entry contains 1641 atoms, of which 757 are hydrogens and 0 are deuteriums.

In the tables below, the ZeroOcc column contains the number of atoms modelled with zero occupancy, the AltConf column contains the number of residues with at least one atom in alternate conformation and the Trace column contains the number of residues modelled with at most 2 atoms.

- Molecule 1 is a protein called Activity-regulated cytoskeleton associated protein 1.

Mol	Chain	Residues	Atoms						ZeroOcc	AltConf	Trace
			Total	C	H	N	O	S			
1	A	83	1531	489	757	137	147	1	0	11	0

There are 4 discrepancies between the modelled and reference sequences:

Chain	Residue	Modelled	Actual	Comment	Reference
A	1	SER	-	expression tag	UNP Q7K1U0
A	2	GLY	-	expression tag	UNP Q7K1U0
A	3	SER	-	expression tag	UNP Q7K1U0
A	4	GLY	-	expression tag	UNP Q7K1U0

- Molecule 2 is water.

Mol	Chain	Residues	Atoms		ZeroOcc	AltConf
			Total	O		
2	A	109	110	110	0	1

3 Residue-property plots (i)

These plots are drawn for all protein, RNA and DNA chains in the entry. The first graphic for a chain summarises the proportions of the various outlier classes displayed in the second graphic. The second graphic shows the sequence view annotated by issues in geometry and electron density. Residues are color-coded according to the number of geometric quality criteria for which they contain at least one outlier: green = 0, yellow = 1, orange = 2 and red = 3 or more. A red dot above a residue indicates a poor fit to the electron density (RSRZ > 2). Stretches of 2 or more consecutive residues without any outlier are shown as a green connector. Residues present in the sample, but not in the model, are shown in grey.

- Molecule 1: Activity-regulated cytoskeleton associated protein 1

Chain A:

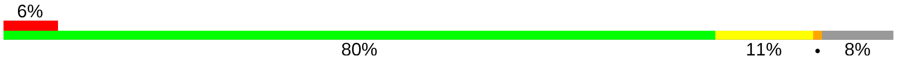

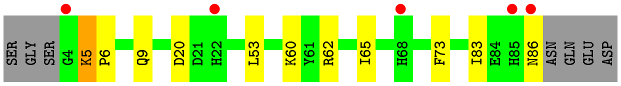

4 Data and refinement statistics (i)

Property	Value	Source
Space group	C 2 2 21	Depositor
Cell constants<br>a, b, c, α, β, γ	37.57Å 51.10Å 82.38Å<br>90.00° 90.00° 90.00°	Depositor
Resolution (Å)	50.00 – 1.05<br>41.19 – 1.05	Depositor<br>EDS
% Data completeness<br>(in resolution range)	92.6 (50.00-1.05)<br>82.2 (41.19-1.05)	Depositor<br>EDS
R_{merge}	(Not available)	Depositor
R_{sym}	0.03	Depositor
$< I/\sigma(I) >$ [1]	1.35 (at 1.05Å)	Xtriage
Refinement program	PHENIX	Depositor
R, R_{free}	0.130 , 0.151<br>0.130 , 0.150	Depositor<br>DCC
R_{free} test set	1633 reflections (5.02%)	wwPDB-VP
Wilson B-factor (Å^2)	11.4	Xtriage
Anisotropy	0.755	Xtriage
Bulk solvent k_{sol}(e/Å^3), B_{sol}(Å^2)	0.38 , 43.3	EDS
L-test for twinning[2]	$< \|L\| > = 0.49$, $< L^2 > = 0.32$	Xtriage
Estimated twinning fraction	No twinning to report.	Xtriage
F_o,F_c correlation	0.98	EDS
Total number of atoms	1641	wwPDB-VP
Average B, all atoms (Å^2)	18.0	wwPDB-VP

Xtriage's analysis on translational NCS is as follows: *The largest off-origin peak in the Patterson function is 15.66% of the height of the origin peak. No significant pseudotranslation is detected.*

[1] Intensities estimated from amplitudes.

[2] Theoretical values of $< |L| >$, $< L^2 >$ for acentric reflections are 0.5, 0.333 respectively for untwinned datasets, and 0.375, 0.2 for perfectly twinned datasets.

5 Model quality (i)

5.1 Standard geometry (i)

The Z score for a bond length (or angle) is the number of standard deviations the observed value is removed from the expected value. A bond length (or angle) with $|Z| > 5$ is considered an outlier worth inspection. RMSZ is the root-mean-square of all Z scores of the bond lengths (or angles).

Mol	Chain	Bond lengths		Bond angles	
		RMSZ	#|Z| >5	RMSZ	#|Z| >5
1	A	0.91	2/803 (0.2%)	0.90	1/1081 (0.1%)

All (2) bond length outliers are listed below:

Mol	Chain	Res	Type	Atoms	Z	Observed(Å)	Ideal(Å)
1	A	86	ASN	CG-ND2	6.01	1.47	1.32
1	A	60	LYS	CB-CG	-5.78	1.36	1.52

All (1) bond angle outliers are listed below:

Mol	Chain	Res	Type	Atoms	Z	Observed(^{o})	Ideal(^{o})
1	A	62	ARG	NE-CZ-NH2	-8.45	116.08	120.30

There are no chirality outliers.

There are no planarity outliers.

5.2 Too-close contacts (i)

In the following table, the Non-H and H(model) columns list the number of non-hydrogen atoms and hydrogen atoms in the chain respectively. The H(added) column lists the number of hydrogen atoms added and optimized by MolProbity. The Clashes column lists the number of clashes within the asymmetric unit, whereas Symm-Clashes lists symmetry related clashes.

Mol	Chain	Non-H	H(model)	H(added)	Clashes	Symm-Clashes
1	A	774	757	739	10	0
2	A	110	0	0	1	0
All	All	884	757	739	10	0

The all-atom clashscore is defined as the number of clashes found per 1000 atoms (including hydrogen atoms). The all-atom clashscore for this structure is 6.

All (10) close contacts within the same asymmetric unit are listed below, sorted by their clash

magnitude.

Atom-1	Atom-2	Interatomic distance (Å)	Clash overlap (Å)
1:A:65:ILE:HD11	1:A:83:ILE:HD12	1.65	0.77
1:A:65:ILE:HD11	1:A:83:ILE:CD1	2.27	0.63
1:A:65:ILE:CD1	1:A:83:ILE:HD12	2.35	0.56
1:A:65:ILE:HG13	1:A:83:ILE:HD11	1.88	0.54
1:A:65:ILE:CD1	1:A:83:ILE:CD1	2.87	0.53
1:A:9[A]:GLN:HG3	2:A:147:HOH:O	2.10	0.50
1:A:53[B]:LEU:HD22	1:A:73:PHE:HE1	1.77	0.50
1:A:65:ILE:CG1	1:A:83:ILE:HD11	2.49	0.42
1:A:5:LYS:HD2	1:A:9[A]:GLN:NE2	2.36	0.41
1:A:6:PRO:HG2	1:A:9[B]:GLN:HG3	2.02	0.41

There are no symmetry-related clashes.

5.3 Torsion angles (i)

5.3.1 Protein backbone (i)

In the following table, the Percentiles column shows the percent Ramachandran outliers of the chain as a percentile score with respect to all X-ray entries followed by that with respect to entries of similar resolution.

The Analysed column shows the number of residues for which the backbone conformation was analysed, and the total number of residues.

Mol	Chain	Analysed	Favoured	Allowed	Outliers	Percentiles	
1	A	92/90 (102%)	92 (100%)	0	0	100	100

There are no Ramachandran outliers to report.

5.3.2 Protein sidechains (i)

In the following table, the Percentiles column shows the percent sidechain outliers of the chain as a percentile score with respect to all X-ray entries followed by that with respect to entries of similar resolution.

The Analysed column shows the number of residues for which the sidechain conformation was analysed, and the total number of residues.

Mol	Chain	Analysed	Rotameric	Outliers	Percentiles	
1	A	87/82 (106%)	85 (98%)	2 (2%)	50	13

All (2) residues with a non-rotameric sidechain are listed below:

Mol	Chain	Res	Type
1	A	5	LYS
1	A	20	ASP

Some sidechains can be flipped to improve hydrogen bonding and reduce clashes. There are no such sidechains identified.

5.3.3 RNA (i)

There are no RNA molecules in this entry.

5.4 Non-standard residues in protein, DNA, RNA chains (i)

There are no non-standard protein/DNA/RNA residues in this entry.

5.5 Carbohydrates (i)

There are no carbohydrates in this entry.

5.6 Ligand geometry (i)

There are no ligands in this entry.

5.7 Other polymers (i)

There are no such residues in this entry.

5.8 Polymer linkage issues (i)

There are no chain breaks in this entry.

6 Fit of model and data (i)

6.1 Protein, DNA and RNA chains (i)

In the following table, the column labelled '#RSRZ> 2' contains the number (and percentage) of RSRZ outliers, followed by percent RSRZ outliers for the chain as percentile scores relative to all X-ray entries and entries of similar resolution. The OWAB column contains the minimum, median, 95th percentile and maximum values of the occupancy-weighted average B-factor per residue. The column labelled 'Q< 0.9' lists the number of (and percentage) of residues with an average occupancy less than 0.9.

Mol	Chain	Analysed	<RSRZ>	#RSRZ>2			OWAB(Å^2)	Q<0.9
1	A	83/90 (92%)	-0.02	5 (6%)	21	21	9, 14, 29, 35	0

All (5) RSRZ outliers are listed below:

Mol	Chain	Res	Type	RSRZ
1	A	86	ASN	5.2
1	A	85	HIS	4.8
1	A	4	GLY	4.2
1	A	22	HIS	3.7
1	A	68	HIS	2.3

6.2 Non-standard residues in protein, DNA, RNA chains (i)

There are no non-standard protein/DNA/RNA residues in this entry.

6.3 Carbohydrates (i)

There are no carbohydrates in this entry.

6.4 Ligands (i)

There are no ligands in this entry.

6.5 Other polymers (i)

There are no such residues in this entry.
